# Generative AI Meets Animal Welfare: Evaluating GPT-4 for Pet Emotion Detection

**DOI:** 10.3390/ani15040492

**Published:** 2025-02-10

**Authors:** Bekir Cetintav, Yavuz Selim Guven, Engincan Gulek, Aykut Asım Akbas

**Affiliations:** 1 Veterinary Faculty, Department of Biostatistics, Burdur Mehmet Akif Ersoy University, Burdur 15030, Türkiye; 2 Institute of Health Science, Burdur Mehmet Akif Ersoy University, Burdur 15030, Türkiye; yavuzselimguven@windowslive.com; 3 Institute of Science, Burdur Mehmet Akif Ersoy University, Burdur 15030, Türkiye; engincan.dev@gmail.com; 4 Veterinary Faculty, Department of Animal Science, Burdur Mehmet Akif Ersoy University, Burdur 15030, Türkiye; aykutaakbas@mehmetakif.edu.tr

**Keywords:** animal emotion detection, dog emotion classification, generative AI, GPT-4, sentiment analysis

## Abstract

Animal emotion detection has emerged as a critical domain in understanding animal welfare and enhancing human–animal interactions. This study evaluates the performance of GPT-4, a generative AI model, in recognizing and classifying pet emotions from images, with a particular focus on dogs. The research was conducted in two phases: a general pet emotion classification across multiple species and a dog-specific classification. In Phase 1, GPT-4 achieved an overall accuracy of 50.2%, reflecting its baseline capability in handling diverse animal images. In Phase 2, accuracy significantly improved to 76.7% due to refined prompts and the use of a targeted dataset. Sentiment analysis of the model’s textual justifications revealed alignment with visual cues in correct predictions and highlighted challenges in ambiguous cases. These findings demonstrate the potential of generative AI in animal emotion detection and emphasize the importance of targeted datasets and advanced prompt engineering. This study contributes to bridging the gap between AI capabilities and practical applications in animal welfare and behavioral research.

## Simple Summary

Understanding how animals feel is essential for improving their welfare and strengthening their bond with humans. In this study, we explored how GPT-4, an advanced AI tool, can detect emotions in pets by analyzing their pictures. Our research had two main steps. First, we tested the AI tool on a variety of animals like cats and rabbits, but the accuracy was moderate. Then, we focused only on dogs, refining the instructions given to the AI tool, which led to much better results. The AI tool was able to recognize emotions like happiness, sadness, and anger in dogs with high accuracy. By examining how the AI tool explained its predictions, we learned about its strengths and where it struggled, especially with emotions that were harder to differentiate. This research shows how AI can help us better understand animals and create tools to support their well-being.

## 1. Introduction

Understanding animal emotions and their expressions has been an enduring focus in animal behavior research. Classical theories, such as Ekman’s basic emotion theory, provided a foundation for identifying primary emotions like fear, anger, and happiness across species [1]. However, recent advancements have highlighted the limitations of these generalized frameworks when applied to non-human animals, especially given the diverse anatomical and behavioral traits across species [2]. Tools like the Facial Action Coding System (FACS) have emerged as pivotal for objectively analyzing facial movements. Originally developed for humans, the FACS has been adapted for various animals, including dogs (DogFACS), cats (CatFACS), and horses (EquiFACS), allowing researchers to decode species-specific facial expressions and associate them with emotional or physiological states [3,4,5]. The emotional states of dogs are often more easily interpretable than those of other domesticated animals, thanks to their unique adaptations and prolonged co-evolution with humans [4]. Compared to species like cats or horses, whose facial expressions are more subtle and often harder for humans to discern, dogs display a wider range of facial signals that align closely with human perceptual cues, making their emotions more readily recognizable [3,5].

The detection of animal emotions has been approached using various methodologies, each with distinct strengths and limitations. Traditional frameworks, like DogFACS, provide a robust foundation for linking facial action units to emotional states, offering interpretable results rooted in human-coded observations [6]. However, these methods require significant manual effort, limiting scalability and their application in real-world, dynamic environments [7,8]. Deep learning (DL)-based systems, such as those leveraging EfficientNet-B0, have demonstrated high accuracy in emotion classification tasks, achieving up to 89% accuracy in controlled settings [9,10,11]. Yet, these systems often struggle with the need for large datasets for fine-tuning, dataset biases, breed-specific morphological variations, and the interpretation of subtle emotions [2,11].

On the other hand, while its performance on animals has not yet been thoroughly evaluated, generative AI models like GPT-4 offer a transformative alternative by addressing some of these challenges through their multimodal capabilities and generalization without task-specific training. Unlike traditional ML/DL-based methods that rely on large, annotated datasets, GPT-4 can integrate visual and textual cues to provide explanations alongside predictions, a feature particularly valuable for non-experts [12,13]. Moreover, the widespread availability of such tools democratizes access to advanced emotion recognition systems, enabling broader applications beyond research labs. However, challenges remain, such as difficulty in detecting micro-expressions and the reliance on textual prompts to guide its analysis, which can introduce variability in performance [14]. Furthermore, the lack of comprehensive evaluations of GPT-4’s performance in animal emotion detection raises questions about its reliability and consistency in this domain. Without systematic empirical studies, its potential remains promising but largely unverified, necessitating caution in its adoption for practical applications [12,15].

In this study, we aim to evaluate the performance of GPT-4, a generative AI tool, in recognizing and classifying pet emotional states from images, positioning this work as a pioneering effort in leveraging generative AI for animal emotion detection. Initially, the model’s capabilities were tested broadly across various pet images without specific prompt engineering to establish a baseline. Subsequently, we focused exclusively on dog images, refining input prompts to improve accuracy and contextual understanding. By analyzing the model’s performance in these two phases, our study sheds light on the potential and limitations of generative AI in this domain, highlighting its role as a versatile and scalable alternative to traditional deep learning models.

To the best of our knowledge, this study represents one of the first systematic efforts to apply a generative AI model like GPT-4 to animal emotion detection. It not only evaluates the feasibility of using generative AI for animal emotion detection but also provides a foundation for future advancements in this interdisciplinary domain.

## 2. Materials and Methods

### 2.1. Data Collection

**Figure 1:**
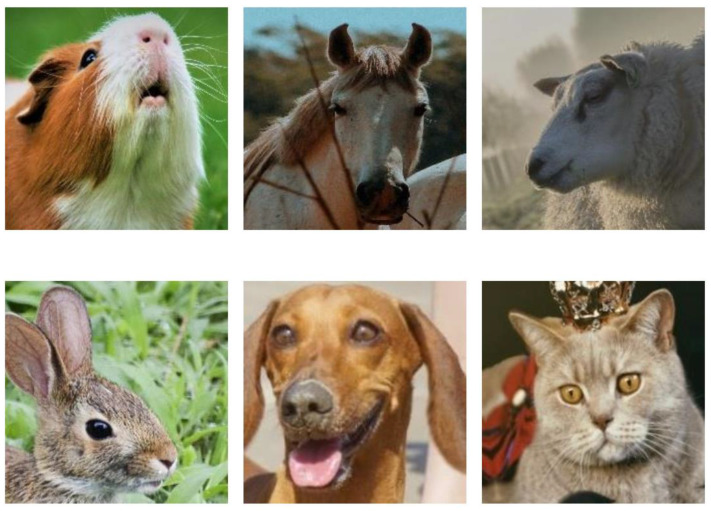
Sample images for general pet/animal emotion classification.

The dataset used in this study was taken from well-known studies by Anwar [16] and Shanbalico [17]. Anwar [16] provides images used to analyze the emotional states of pet animals. The dataset was retrieved from the Kaggle Repository (Pet’s Facial Expression Image Dataset), a platform that provides open access to datasets under the Creative Commons Attribution 4.0 International (CC BY 4.0) license. The dataset features various animal species, including dogs, cats, rabbits, hamsters, sheep, horses, and birds. These images capture three main emotional states (happiness, sadness, and anger), providing a valuable resource for understanding and examining variations in animals’ facial expressions (Figure 1). A total of 750 images of different animals were evaluated under a simplified analysis scenario where only emotional state predictions were recorded.

Shanbalico [17] offers a dataset specifically focusing on dogs’ emotional states. The “Dog Emotion” dataset, retrieved from the Kaggle Repository, consists of 4000 images of dogs, with equal representation of four emotional states: angry, happy, relaxed, and sad. The dataset, licensed under CC0: Public Domain, was manually annotated to ensure accuracy. From this dataset, 1517 images were carefully selected and validated through expert reviews by licensed veterinary professionals. This rigorous selection process ensures the reliability of the data used in this study to analyze canine emotional states. In this group, the model was tasked with not only predicting the emotional state but also providing detailed explanations of the visual cues underpinning its predictions.

### 2.2. Experimental Setup

This study was conducted through a two-phased experimental process: General Pet Emotion Classification and Dog-Specific Emotion Classification. Each phase was designed to evaluate the model’s performance and explainability by focusing on different analytical objectives.

#### 2.2.1. General Pet/Animal Emotion Classification

In the first phase, a general emotion classification was performed on 750 pre-labeled pet images from the dataset. During this phase, the model was tasked with predicting the emotional states of the animals in the images as “Happy”, “Sad”, or “Angry”. Only basic predictions were required, allowing for a straightforward evaluation of the model’s performance. The baseline performance obtained in this phase was used to measure the model’s overall accuracy across a diverse range of animal species and its success rate for each emotional category. The simple prompt used during this phase was as follows: “*Please guess the emotional state of the animal in this picture. Indicate whether it is ‘happy’, ‘angry’, or ‘sad’ based on the features visible in the image*”. This minimalistic approach enabled the model to produce results quickly, facilitated the analysis of baseline performance, and also allowed GPT to be “unsure”.

#### 2.2.2. Dog Emotion Classifications and Sentiment Analysis

In the second phase, the analysis focused exclusively on 1517 dog images. During this phase, the model was required to provide greater explainability. To enable a more detailed examination of facial expressions specific to dogs, the model was instructed to justify its predictions by explaining the observed emotional states. The detailed prompt used in this phase was as follows: *“I will provide you with images of dogs classified into one of four categories: angry, happy, comfortable, or sad. Your task is to analyze each photo and make a single-word prediction based on these classes. Additionally, explain your reasoning for the prediction by describing the visual cues observed in the image”.*

Prompt engineering in this phase allowed for the evaluation of not only the accuracy of the model’s predictions but also the reasoning behind them. The model’s predictions were based on visual features, such as facial expressions and body language, specific to dogs. Detailed explanations were analyzed for their alignment with the predicted emotional categories and the consistency of the visual cues used.

The other significant aspect of the second phase focuses on analyzing the expressions used in the model’s predictions. Through sentiment analysis, the keywords and expressions appearing in the model’s predictions were examined in detail and assessed for their associations with specific emotional categories. This analysis provided a critical foundation for understanding the model’s ability to accurately perceive emotional states from animals’ facial expressions. During the sentiment analysis process, expressions from correct and incorrect predictions were analyzed separately. These expressions were classified by comparing them against a predefined list of keywords. For example, words directly indicating emotional states, such as “happy”, “sad”, “relaxed” and “angry”, were evaluated for consistency with the predicted category. Additionally, terms representing animal behaviors, such as “bark”, “wag”, “growl”, and “smile”, were included in the analysis. Furthermore, keyword frequency analyses were conducted, and the most frequently used words were presented as bar charts. These analyses provided insights into the features that stood out in the model’s correct predictions and the expressions that predominated in its errors.

### 2.3. Generative AI Model and Evaluation Metrics

OpenAI’s GPT-4o model was utilized to analyze and classify the emotional states of pets based on their facial expressions. The analysis leveraged the datasets and evaluated the model’s performance under given scenarios. The images underwent preprocessing using Python and were converted into base64 format for analysis by OpenAI’s GPT-4o model. The model’s predictions and detailed outputs were stored in structured datasets for further analysis. Results were assessed using error matrices and keyword analysis to visualize the model’s accuracy and errors.

In the context of multimodal analysis, specialized metrics were employed to evaluate the alignment between visual and linguistic features used in the model’s predictions. For instance, in the detailed analysis scenario, the consistency of the model’s explanations with the predicted emotional states was assessed. Additionally, confusion matrices were utilized to analyze the distribution of true positives, false positives, and false negatives for each emotional category. The key metrics used to evaluate the model’s overall performance include:

**Accuracy**: To measure the overall correctness of the predictions.

(1) $$Accuracy=\frac{True\;Positives+True\;Negatives}{Total\;Instances}$$

**Precision**: To evaluate the proportion of correctly predicted instances for each class.

(2) $$Precision=\frac{True\;Positives}{True\;Positives+False\;Positives}$$

**Recall**: To assess the model’s ability to identify all relevant instances of each class.

(3) $$Recall=\frac{True\;Positives}{True\;Positives+False\;Negatives}$$

**F1 Score**: To provide a harmonic mean of precision and recall, offering a balanced evaluation of model performance, particularly in cases of class imbalance.

(4) $$F1\;Score=2\times \frac{Precision\times Recall}{Precision+Recall}$$

In the context of multimodal analysis, specialized metrics were employed to evaluate the alignment between visual and linguistic features used in the model’s predictions. For instance, in the detailed analysis scenario, the consistency of the model’s explanations with the predicted emotional states was assessed. Additionally, confusion matrices were utilized to analyze the distribution of true positives, false positives, and false negatives for each emotional category.

## 3. Results

### 3.1. Phase 1—General Pet Emotion Classification Results

In the first phase, GPT-4 was evaluated on a diverse dataset of 750 images representing various animal species, including cats, rabbits, birds, and others. The goal of this phase was to establish a baseline for the model’s performance across multiple species using minimalistic prompts. The results (Table 1) showed moderate performance, reflecting the complexity of generalizing emotional states across species with varied facial expressions and physical traits.

The model achieved an overall accuracy of 50.2%, with additional performance metrics revealing the nuances of its predictions. The precision was 55.6%, indicating that slightly more than half of the positive predictions were correct. The recall score of 51.2% highlighted the model’s moderate capability to identify relevant instances across all categories. Finally, the F1 Score was 53.3%, representing a harmonic mean of precision and recall, demonstrating a balance between correctly identifying emotional states and minimizing false predictions.

**Figure 2:**
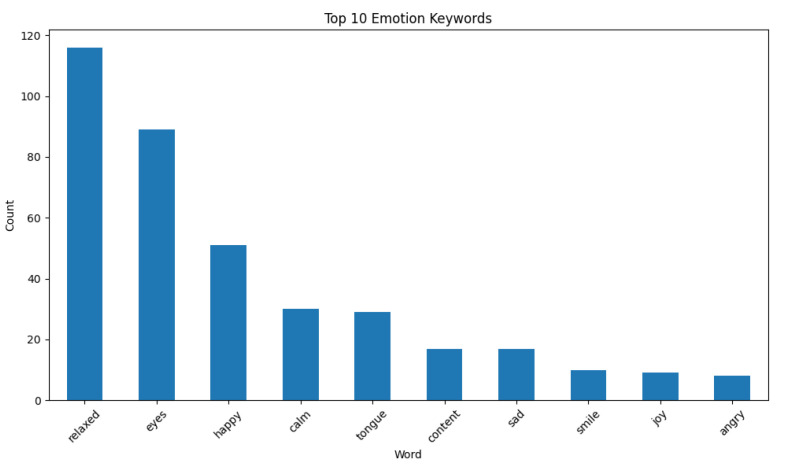
Sentiment analysis results for the top 10 emotion keywords.

Among the three emotional categories (“Happy”, “Sad”, and “Angry”), the model demonstrated the highest performance in predicting “Happy”, suggesting that positive emotional states were more distinguishable based on visual cues (Figure 2). However, predictions for the “Sad” and “Angry” categories showed lower accuracy, likely due to the subtle and species-specific nuances of these emotions. These results indicate that while the model provides a baseline understanding of general pet emotions, its performance is constrained by the dataset’s diversity and the simplicity of the prompts used.

### 3.2. Phase 2—Dog Emotion Classification Results

The second phase of the study focused exclusively on 1517 images of dogs, aiming to assess GPT-4’s ability to classify emotions with greater specificity and provide detailed justifications for its predictions. Enhanced prompts were utilized to instruct the model to analyze visible features, such as facial expressions and body language, and offer explanations for its predictions.

The confusion matrix presented in Table 2 provides a comprehensive breakdown of the model’s classification performance for four emotional states: Angry, Happy, Relaxed, and Sad. Among the correctly classified instances, the model demonstrates exceptional performance for the Happy class with 331 accurate predictions, followed by Relaxed with 292 correct classifications. The Angry class was also accurately detected, achieving 254 accurate predictions. However, certain misclassifications are noteworthy: 91 Happy instances were predicted as Angry, and 111 Relaxed instances were misclassified as Sad. Furthermore, there was minor confusion between Relaxed and Happy labels, with 47 Relaxed instances misclassified as Happy. These results indicate that while the model is adept at identifying distinct emotional states, overlapping features among certain classes (e.g., Relaxed and Sad) introduce ambiguity, leading to classification errors.

The model’s performance metrics further validate its effectiveness. An overall accuracy of 76.6% demonstrates that the majority of predictions are correct. Precision (76.8%) highlights the model’s ability to reduce false positives, with the highest precision observed for the Happy and Relaxed classes. Recall (78.8%) indicates that the model is particularly effective at identifying true positives, with outstanding recall for the Angry class (93.7%), signifying the model’s robustness in detecting this state. The F1 Score (76.7%) reflects a balanced trade-off between precision and recall across all classes, underscoring the model’s general reliability.

**Figure 3:**
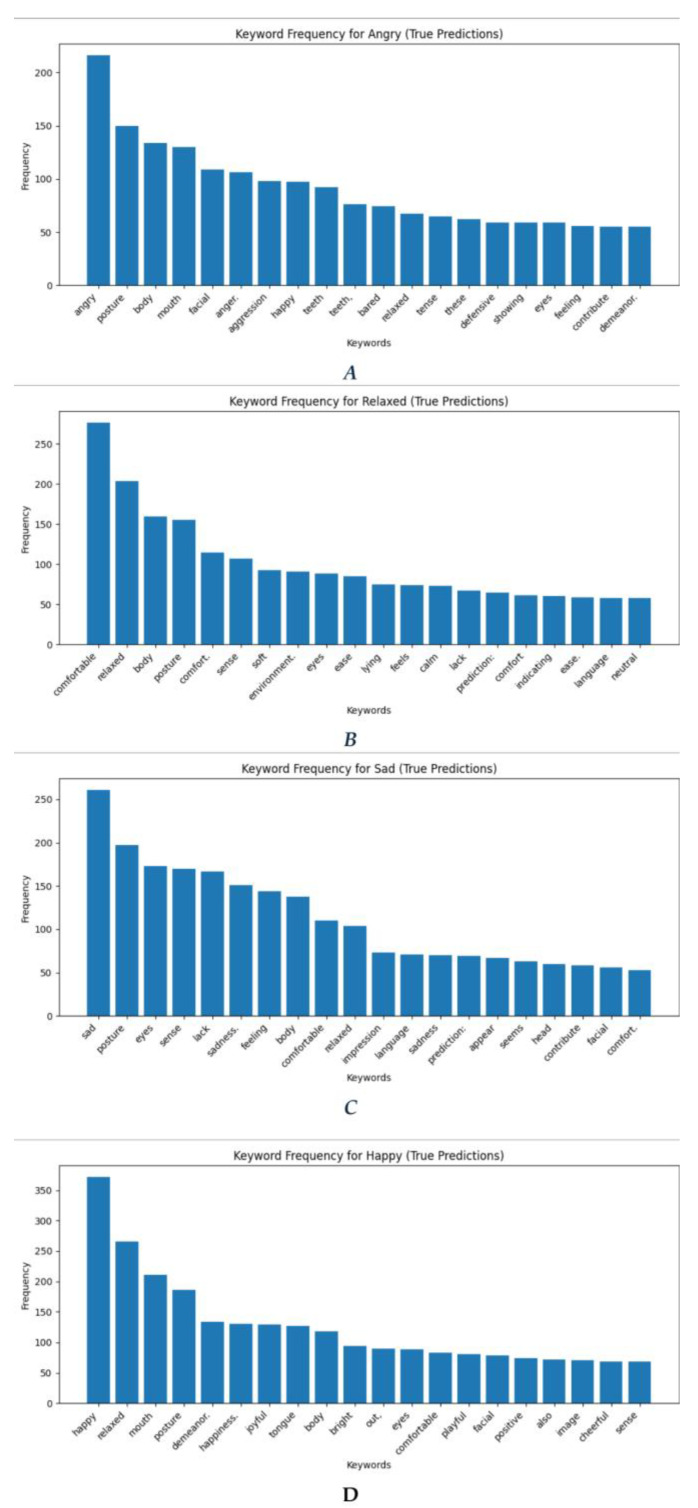
Word clouds generated based on *true* predictions of angry (**A**), relaxed (**B**), sad (**C**) and happy (**D**) emotional states.

A crucial aspect of Phase 2 involved conducting a sentiment analysis of the language patterns and keywords used by GPT-4 in its predictions. This analysis aimed to evaluate the model’s reasoning and its ability to align textual explanations with observable visual cues in the images. The keyword frequency analysis for true predictions (Figure 3) across the four emotional states (Angry, Happy, Relaxed, and Sad) highlights key linguistic patterns associated with each sentiment. These findings provide insights into the most salient features that contributed to the accurate classification of each emotional state.

***Angry Predictions:*** In the Angry sentiment (Figure 3A), the most frequently occurring keywords are “angry”, “posture”, “body”, and “mouth”. These keywords are indicative of physical expressions and body language that are strongly associated with anger, such as aggressive postures or facial expressions. Additional terms like “facial” and “aggression” further emphasize the importance of visible cues in predicting anger. The presence of words like “happy” and “relaxed”, albeit at lower frequencies, likely reflects phrases such as “looks not happy” or “not relaxed”, indicating a lack of these states rather than their presence. This subtle nuance in the dataset could explain their appearance and reduce concerns about potential misclassifications.

***Relaxed Predictions:*** In the Relaxed sentiment (Figure 3B), the most frequent keywords include “comfortable”, “relaxed”, “body”, and “posture”. These words signify physical and emotional ease, which are strong indicators of relaxation. Words like “comfort”, “sense”, and “environment” further highlight the importance of external surroundings and physical states in defining this emotional category. The occurrence of terms such as “lying” and “calm” suggests behavioral attributes that reinforce the relaxed state, differentiating it from other sentiments like Happy or Sad.

***Sad Predictions:*** For the Sad sentiment (Figure 3C), the keywords “sad”, “posture”, and “eyes” emerge as the most frequent, pointing to observable physical cues associated with sadness. Other prominent terms, such as “lack”, “feeling”, and “sadness”, underline the emotional and behavioral components of this sentiment. Interestingly, terms like “comfortable” and “relaxed” appear at lower frequencies, suggesting occasional ambiguity or overlap in the dataset. The presence of words like “impression” and “language” indicates that subtler cues, possibly linguistic or contextual, play a role in predicting sadness.

***Happy Predictions:*** For the Happy sentiment (Figure 3D), the keywords “happy”, “relaxed”, and “mouth” dominate the true predictions. This suggests that the model associates happiness with positive facial expressions and an overall sense of relaxation. Terms like “demeanor”, “happiness”, and “joyful” reinforce the link between outward expressions of joy and the label. Additionally, keywords such as “tongue”, “bright”, and “playful” indicate the presence of unique behavioral features tied to happiness, such as playfulness or cheerful demeanor.

***Comparative Observations:*** Angry and Happy sentiments rely heavily on physical and facial expressions, with keywords like “mouth” and “posture” being common to both. However, aggression-related terms (e.g., “anger” and “aggression”) are unique to Angry, while cheerful terms (e.g., “joyful” and “bright”) define Happy. Relaxed predictions are strongly associated with comfort and environmental factors, distinguishing it from other emotional states. Sad predictions emphasize emotional depth and lack of vitality, often conveyed through physical cues like “posture” and “eyes”.

### 3.3. Discussion of Successful and Failed Cases

To better understand the model’s decision-making process for predicting emotional states, we analyzed a series of predictions through both qualitative observations and a DogFACS-based perspective. The first image (Table 3A) demonstrates a correct prediction (Angry), where the model accurately identified clear visual cues of aggression. Key features such as bared teeth, pulled-back ears, and tense posture align with DogFACS Action Units like AU110 (Upper Lip Raise), AU145 (Ears Pulled Back), and AU25 (Lips Parted), which are strong indicators of an angry emotional state. The model’s focus on these biologically relevant markers resulted in a correct classification.

The second image (Table 3B), however, shows an incorrect prediction (labeled “Angry”, predicted “Happy”). Here, the model misinterpreted the open mouth and wide eyes as signs of playfulness or joy rather than aggression. The fur styling and relaxed appearance may have further contributed to the model’s bias toward classifying the image as Happy. From a DogFACS perspective, critical indicators of anger, such as AU110 (Upper Lip Raise) or AU145 (Ears Pulled Back), are absent, which the model may have overlooked. This error highlights the challenge of distinguishing between subtle differences in expressions, such as a panting smile versus an aggressive snarl. The third image (Table 3C) represents another correct prediction (labeled “Happy”, predicted “Happy”) and showcases the model’s ability to recognize joyful emotional states. The puppy exhibits a relaxed body posture and stands in a playful position, with its tongue out, corresponding to AU25 (Lips Parted) and AU27 (Tongue Show), which are often associated with excitement and contentment in dogs. Additionally, the bright environment with lush green grass further reinforces the cheerful context, aiding the model in making an accurate classification.

## 4. Discussion

The findings of this study reveal significant insights into the applicability of generative AI tools like GPT-4o for animal emotion detection, particularly in the context of pet and dog-specific classifications. This discussion contextualizes the results within the broader landscape of emotion recognition research, addressing the implications, limitations, and potential future directions.

### 4.1. General Pet Emotion Classification

Our findings in Phase 1, where GPT-4 achieved an overall accuracy of 50.2%, reflect challenges similar to those observed in the human ability to recognize emotions in animals. Prior research has shown that humans often excel at identifying emotions such as happiness and anger but struggle with sadness and fear, achieving below 50% accuracy for these categories [18]. Another study demonstrated that posture analysis using DeepLabCut yielded 60–70% accuracy, emphasizing the difficulty of cross-species emotion recognition [19]. This is partly due to variations in anatomy, behavioral expressions, and environmental contexts, as seen in the study by Franzoni et al. [7], who achieved improved accuracy by tailoring datasets to specific breeds and environmental factors.

The model in this study excelled in detecting “Happy” emotions, likely due to distinct visual cues such as playful postures and relaxed appearances, as reported in studies like those of Boneh-Shitrit et al. [6] and Bhave et al. [11], where clear positive expressions are easier to identify than subtle or overlapping emotions such as sadness or fear. The struggles with “Angry” and “Sad” classifications underscore the limitations of generic models in distinguishing subtle emotional markers, echoing the findings of Mendl et al. [20] on the importance of context and dimensional approaches in emotion analysis.

### 4.2. Dog-Specific Emotion Classification

The shift to a dog-specific dataset in this study resulted in a significantly improved accuracy of 76.6%, demonstrating the value of species-specific data and tailored analytical approaches. Franzoni et al. [11] similarly observed enhanced performance, achieving over 89% accuracy by integrating DogFACS with advanced deep learning techniques like EfficientNet-B0, which effectively captured facial and postural nuances. In contrast, Bhave et al. [11] reported a more modest accuracy of 74.32% using ResNet50, which suggests that architecture choice and dataset design are critical for optimizing model performance.

The use of DogFACS to interpret facial and postural cues, such as AU110 (Upper Lip Raise) and AU145 (Ears Pulled Back) for anger, provided biologically relevant insights into emotion recognition, consistent with studies by Boneh-Shitrit et al. [6], who emphasized DogFACS variables in explaining positive anticipation and frustration. However, misclassifications, such as interpreting a panting dog as “Happy” instead of “Relaxed”, revealed the model’s difficulty with nuanced distinctions, echoing the challenges identified by Ferres et al. [19] when relying solely on visual cues.

### 4.3. Insights from Comparative Emotion Detection

The stark performance difference between Phase 1 (general pet emotion classification) and Phase 2 (dog-specific classification) aligns with findings in the literature. Specifically, the improved accuracy in Phase 2 (87.7%) supports the hypothesis that dogs’ evolutionary proximity to humans contributes to their better emotional expressiveness and recognizability. As Burza et al. [21] suggest, shared experiences between species enhance the ability of humans—and by extension, AI—to recognize heterospecific emotions. Dogs, having evolved alongside humans, exhibit what are commonly referred to as primary emotions, such as happiness and sadness, which are more stimulus-bound and evolutionarily adaptive [18].

Dogs present a unique comparative model for emotion studies due to their homologous facial anatomy with humans [22] and shared mammalian neuroanatomy for basic emotions, such as happiness and anger [23]. Their facial cues and expressions are particularly well-suited for emotion detection by both humans and AI systems. In this study, the refinement of prompts and the use of a dog-specific dataset enabled GPT-4 to leverage these traits effectively, resulting in significantly improved performance metrics compared to Phase 1. This outcome reinforces the importance of domain-specific datasets and highlights dogs as an ideal species for advancing research in animal emotion detection [24].

### 4.4. Limitations, Challenges, and Future Directions

While the study demonstrates the potential of GPT-4 in animal emotion detection, it also highlights critical limitations. The model occasionally struggled with nuanced or overlapping emotional states, a challenge previously identified in multimodal AI applications. Moreover, the general pet dataset in Phase 1 exposed difficulties in generalizing across species with distinct facial expressions and body language. These findings echo challenges reported in cross-species emotion recognition studies.

Additionally, while the study utilized a relatively large dataset in the dog-specific phase (1517 samples), which significantly contributed to the model’s improved accuracy and robustness, the general pet dataset (750 samples) presented unique challenges. Specifically, the large variety of species included in the general pet dataset resulted in relatively fewer samples per species, which may have limited the model’s ability to generalize effectively across all animals. Considering the inherent difficulties of recognizing emotions in non-dog species, this data distribution poses a restriction on the generalizability of findings in the pet phase of the study. Future research could benefit from species-specific studies, where datasets are curated individually for each animal type, enabling more robust and reliable emotion recognition tailored to the unique expressions and behaviors of each species. Additionally, exploring domain-specific fine-tuning techniques could further optimize generative AI models like GPT-4o for specific animal categories, enhancing their performance and accuracy. Another promising direction involves the integration of multimodal data, such as audio signals and contextual environmental information, to provide a more comprehensive understanding of animal emotions. This multimodal approach could help capture subtle emotional nuances that are difficult to identify from visual data alone.

## 5. Conclusions

By evaluating GPT-4’s performance in general and species-specific contexts, this study contributes to the growing field of generative AI-driven animal emotion detection. The findings underscore the importance of targeted datasets and refined prompts in advancing the applicability of generative AI tools. While challenges remain, this research lays the groundwork for future advancements, bridging the gap between generative AI capabilities and practical applications in animal welfare and behavioral understanding.

## Figures and Tables

**Table 1:** Confusion matrix and performance metrics of Phase 1 of the prediction model.

	Labeled “Angry”	Labeled “Happy”	Labeled “Sad”
Predicted “Angry”	96	3	9
Predicted “Happy”	147	235	188
Predicted “Sad”	2	9	47
Predicted “Unsure”	5	3	6
Precision = 0.556			
Recall Score = 0.512			
F1 Score = 0.533			
Accuracy Score = 0.502			

**Table 2:** Confusion matrix and performance metrics of Phase 2 of the prediction model.

	Labeled “Angry”	Labeled “Happy”	Labeled “Relaxed”	Labeled “Sad”
Labeled “Angry”	254	3	6	8
Labeled “Happy”	91	331	7	4
Labeled “Relaxed”	18	47	292	111
Labeled “Sad”	10	0	50	284
Precision = 0.768				
Recall Score = 0.788				
F1 Score = 0.767				
Accuracy Score = 0.766				

**Table 3:** Illustration of some correct and incorrect predictions.

(**A**)		
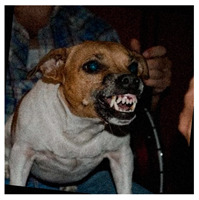	Labeled “***Angry***” Predicted “***Angry***”	This prediction is based on the dog’s facial expression and body language. The dog has bared teeth, which is a typical sign of aggression or anger. Its ears may be pulled back tightly, and the overall posture suggests it is feeling threatened or upset. Additionally, the tense muscles and glaring eyes further reinforce the idea of anger.
(**B**)		
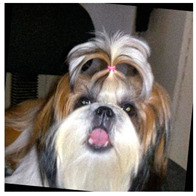	Labeled “***Angry***” Predicted ***“Happy”***	The prediction of “Happy” is based on the dog’s open mouth, which looks like it might be smiling or playfully panting. The wide eyes and relaxed facial expression contribute to this impression of joy and contentment. Additionally, the overall posture and fur style suggest a well-groomed and cared-for pet, which often reflects a positive emotional state.
(**C**)		
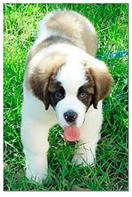	Labeled “***Happy***” Predicted ***“Happy”***	The puppy appears to have a relaxed body posture and is standing in a playful position. Its tongue is out, which often indicates excitement and a jovial mood in dogs. The lush green grass adds to the cheerful atmosphere, suggesting that it is enjoying its environment. Overall, these visual cues suggest a state of happiness.

## Data Availability

The datasets used in this study were retrieved from the Kaggle Repository, a platform offering open access to datasets. The first one was titled *Pet’s Facial Expression Image Dataset* and was under the Creative Commons Attribution 4.0 International (CC BY 4.0) license. The dataset can be accessed at https://www.kaggle.com/datasets/anshtanwar/pets-facial-expression-dataset (accessed on 10 December 2024). The second one, the “Dog Emotion” dataset, retrieved from the Kaggle Repository, consists of 4000 images of dogs The dataset, licensed under CC0: Public Domain, was manually annotated to ensure accuracy. The dataset can be accessed at https://www.kaggle.com/datasets/danielshanbalico/dog-emotion (accessed on 15 January 2025).
